# Properties of Arabinogalactan Proteins (AGPs) in Apple (*Malus* × *Domestica*) Fruit at Different Stages of Ripening

**DOI:** 10.3390/biology9080225

**Published:** 2020-08-14

**Authors:** Agata Leszczuk, Justyna Cybulska, Tomasz Skrzypek, Artur Zdunek

**Affiliations:** 1 Institute of Agrophysics, Polish Academy of Sciences, Doświadczalna 4, 20-290 Lublin, Poland; j.cybulska@ipan.lublin.pl (J.C.); a.zdunek@ipan.lublin.pl (A.Z.); 2 Confocal and Electron Microscopy Laboratory, Centre for Interdisciplinary Research, John Paul II Catholic University of Lublin, Al. Kraśnicka 102, 20-718 Lublin, Poland; tskrzypek@kul.pl

**Keywords:** arabinogalactan proteins, calcium, cell wall, fruit, ripening process, structural properties

## Abstract

Arabinogalactan proteins (AGPs) are constituents of the cell wall–plasma membrane continuum in fruit tissue. The aim of the study was to characterise AGPs contained in fruit by determination of their chemical structure and morphological properties. The results were obtained from in and ex situ investigations and a comparative analysis of AGPs present in *Malus* × *domestica* fruit at different stages of ripening from green fruit through the mature stage to over-ripening during fruit storage. The HPLC and colorimetric methods were used for analyses of the composition of monosaccharides and proteins in AGPs extracted from fruit. We have found that AGPs from fruit mainly consists of carbohydrate chains composed predominantly of arabinose, galactose, glucose, galacturonic acid, and xylose. The protein moiety accounts for 3.15–4.58%, which depends on the various phases of ripening. Taken together, our results show that the structural and morphological properties of AGPs and calcium concentration in AGPs are related to the progress of ripening, which is correlated with proper fruit cell wall assembly. In line with the existing knowledge, our data confirmed the typical carbohydrate composition of AGPs and may be the basis for studies regarding their presumed properties of binding calcium ions.

## 1. Introduction

Arabinogalactan proteins (AGPs), i.e., elements of the plant extracellular matrix localised between the cell wall and plasma membrane, are characterised by several extraordinary properties. First, AGPs are structural proteins belonging to hydroxyproline-rich glycoproteins (HRGPs), which represent one of the most frequently post-translationally modified groups of proteins. The protein moiety constitutes a maximum of 10% of the total AGP molecule, and it is predominantly made of carbohydrate chains. Extensive glycosylation of AGPs beginning in the endoplasmic reticulum and Golgi apparatus is related to the occurrence of specific glycosyltransferases. Structural analysis of AGPs extracted from various plant organs from various plant species revealed that heavy glycosylation of AGPs mainly consists of arabinose (Ara-) and galactose (Gal-) rich polysaccharide side chains attached to the AGP core protein [1,2,3]. Detailed knowledge of the AGP molecule composition was provided by analyses of the aerial part of *Echinacea purpurea*, juice, and suspension culture, in which AGPs were composed of a high amount of polysaccharides (83%), some uronic acids (4–5%), and low protein content (7%). The monosaccharide analysis of isolated AGP showed that Gal and Ara comprised over 90% of the monosaccharide constituents of the purified AGP, with minor amounts of glucuronic acid (GlcA), and rhamnose (Rha). The protein moiety is typically rich in hydroxyproline, serine, and threonine and covalently linked to carbohydrate moieties [4,5]. In the case of fruit, the chemical composition of AGPs is quite similar, and the sugar part is dominant, while the core protein constitutes only a few percent of their total molecular mass. It has been found that AGPs extracted from *Lycium chinense* fruit tissues are composed of Ara and Gal at a ratio of 3:1 with a small amount of fucose (Fuc), xylose (Xyl), mannose (Man), glucose (Glc), and galacturonic acid (GalA). The protein moiety represents only 3–5% of the AGP molecule and the total amino acid residues mainly contain serine, proline, and glutamic acid [6]. AGPs extracted from apple [7] and pear [8] juices are characterised by a similar chemical composition, i.e., glycosidic linkages with dominance of Gal and L-Ara residues and a small amount of the protein moiety. The importance of heavy glycosylation of AGPs to the AGP function was a subject of numerous studies, in which AGPs were implicated in various processes e.g., cell proliferation, cellular signalling, pollen tube targeting during the progamic phase in plant development, programmed cell death, organ abscission, and interactions with plant microbes and plant regulators [9]. Moreover, as cell wall mechanic modulators, AGPs are involved in cell assembly by crosslinking with other cell wall components. They are an important part of the APAP1 complex, in which they are covalently attached to cell wall matrix polysaccharides, i.e., hemicellulose and pectins, via their arabinogalactan decorations [10,11,12]. 

Another typical feature of AGPs differentiating them from pectins is their spatiotemporal distribution, which is correlated with their chemical composition, and exactly with the appearance of the glycosylphosphatidylinositol lipid anchor (GPI) at the C-terminus of the AGP molecule [9]. AGPs are anchored to the plasma membrane by GPI, while their sugar chains are located in the cell wall and spaces between the cell wall and plasma membrane. Thus, AGPs are candidates as plasma membrane–cell wall anchors that may be involved in cell adhesion, acting as mechanosensors and receptors of pathogen effectors [13,14]. Moreover, the possibility to process AGPs by endogenous phospholipase C or D allows release of AGPs from the plasma membrane to the cell wall or extracellular destinations [1]. 

However, the mechanism and factors taking part in changes in AGP localisation in cell are still unknown. One of the presumable actions connected with the distribution of AGPs is their interlinking with calcium ions depending on the bonds of glucuronic acids residues of arabinogalactan (AG) chains and Ca^2+^ [15]. Lamport and co-workers [16] analysed AGP as a periplasmic Ca^2+^ capacitor involved in intramolecular Ca^2+^ binding and assumed that the dynamic Ca^2+^ is recycled by AGP-Ca^2+^ in many biological processes. The structural and physical properties of fruit tissue are determined among others by polysaccharide–calcium cross-links. Metabolic changes in Ca^2+^ in the cell wall and in an apoplastic solution are related to cell wall and plasma membrane stabilisation [17].

The goal of our study is to characterise AGPs contained in fruit by determination of their morphological properties and chemical structure. The aims were achieved through in and ex situ investigations and a comparative analysis of AGPs present in *Malus* × *domestica* fruit at different stages of ripening from green fruit through the mature stage to over-ripening during fruit storage. To date, the knowledge of the features of AGPs from fruit has been provided by a limited number of studies, and none of them reported changes occurring during ripening stages. The extensive glycosylation of AGPs, their unusual localisation in cells, the presence of an anchor, connections with plasma membrane, and interlinkage with other cell wall components emphasise their importance and prompt the need to undertake research on their participation in the fruit ripening process.

## 2. Material and Methods

### 2.1. Material

Apple (*Malus* × *domestica*) fruit cv. “Jonaprince” were purchased from a local producer, and used for the experiments. Apple fruit at three different stages were chosen: before the ripening process, a month before the optimal harvest date—the immature green stage (‘green fruit’), at the full red ripe stage, at the optimum maturity for this cultivar (‘red fruit’)—and after 1 month of storage for observation of the over-ripening process during storage (‘stored fruit’). The apple fruit were stored in a cold room at 2 °C in a normal atmosphere. Fruit with similar features and without visible symptoms of disease and bruising were chosen. For the isolation procedure, 30 g of homogenised apple tissue from 5 fruit at the mentioned ripening stages were used. To carry out microscopic analyses, 5 apple fruits for each stage were analysed. The 10 cube-shaped samples were obtained by excision of the external part of the fruit, which included the epidermal and hypodermal layers, and parenchyma from a depth of ca. 0.5 cm under the skin.

### 2.2. Isolation of AGPs from Fruit

AGPs were extracted from apple fruit at different stages of ripening according to the protocol developed by Lamport [18], based on the selective precipitation of AGPs with the β-D-Glucosyl Yariv reagent (Biosupplies, Australia) [19]. Frozen fruit parenchymal tissue was homogenised and stirred in 2% CaCl_2_ for 3 h at room temperature. After centrifugation for 30 min, the supernatant was mixed with an equal volume of Yariv reagent (1 mg mL^−1^) in 2% (*w/v*) CaCl_2_ (Biosupplies, Australia). After precipitation overnight at room temperature, the precipitate (insoluble Yariv-AGP complex) was resuspended in MiliQ water. For reduction in diazo linkages, Na metabisulphite was added and heated at 50 °C until the suspension was decolourised. After obtaining a clear yellow solution, the suspension was transferred into a dialysis bag with a 12 kDa MW cut-off (32 mm flat width, Sigma, St. Louis, MO, USA) and stirred overnight at room temperature. The dialyzate was freeze-dried.

### 2.3. Morphology Imaging of AGPs

Lyophilised AGPs extracted from fruit at different stages of ripening were imaged in high vacuum (5 × 10^−3^ Pa), using a secondary electron detector at 3 kV with a scanning electron microscope (SEM, Zeiss Ultra Plus, Oberkochen, Germany).

### 2.4. Quantification of Monosaccharide Compositions of AGPs

The monosaccharide composition was determined according to the method previously reported by Lv [20] and Zhang [21] with some modifications. In order to decompose AGP molecules, approximately 2 mg of each AGP was subjected to methanolysis at 80 °C for 72 h and hydrolysis with 2 mL of 3 M trifluoroacetic acid (TFA) solution at 100 °C for 72 h. Next, the samples were lyophilised. Derivatisation of monosaccharides with 1-phenyl-3-methyl-5-pyrazolone (PMP) was then carried out by adding 1 mL of water, 50 µL of 0.3 M NaOH, and 50µL of a 0.5 M solution of PMP in methanol. The mixture was incubated at 70 °C for 60 min and cooled; next, 50 µL of 0.3 M HCl was added. The resulting solution was extracted three times with 1 mL of chloroform and finally filtered through a 22 µm membrane. Standards of the monosaccharides (Ara, Fuc, Gal, GalA, Glc, GlcA, Man, Rha, and Xyl) were treated in the same way as the AGP samples.

The PMP-labelled samples were analysed using an HPLC system consisting of an 1130 HPLC quaternary pump, an S 5300 sample injector, an S 4120 column oven, and an S 3350 PDA Detector (Sykam GmbH, Gewerbering, Germany) equipped with a Zorbax Eclipse XDB-C18 (4, 6 mm i.d. × 250 mm, 5 µm) analytical column coupled with an Agilent Eclipse XDB-C18 guard column (12.5 × 4.6 mm i.d., 5 μm). The mobile phase was composed of 0.1 M phosphate buffer (pH 6.7) and a 50% solution of 0.1 M phosphate buffer in acetonitrile at a ratio of 69:31% (*v/v*) in the isocratic elution mode. The injection volume was 20 μL, the flow rate was 1.8 mL/min at 30 °C, and the detection wavelength was 246 nm.

### 2.5. Quantification of Uronic Acids in AGPs

Uronic acids (UA) content in AGPs was determined with a San++ 140 Continuous Flow Analyzer (Skalar, Breda, The Netherlands) according to the colorimetric method used by Blumenkrantz and Asboe-Hansen [22] and according to the procedure modified previously by Cybulska [23]. Briefly, the samples were hydrolysed with 13 M sulphuric acid at 25 °C for 90 min; next, the sample was decomposed in 96% sulphuric acid with di-sodium tetraborate. For UA content measurement, the products were transformed into furfuric derivatives by reaction with the 3-phenyl phenol and the sodium hydroxide to form a coloured dye, which was measured at 530 nm. Mono-galacturonic acid solutions were used to create a standard calibration curve.

### 2.6. Quantification of Protein Components in AGPs

The protein content was estimated with the Bradford method [24] with Bovine Serum Albumin (BSA) as a standard.

### 2.7. Quantification of Calcium Content in AGPs

The calcium content in all examined fruit samples was determined with the colorimetric method using a San++ 140 Continuous Flow Analyzer (Skalar, Breda, The Netherlands) according to the procedure described previously by Blumenkrantz and Asboe-Hansen [22] and modified by Mierczyńska [25]. The procedure is based on the following reactions: decomposition with a di-sodium tetraborate solution (1), transformation into furfural derivatives reacted with 3-phenylphenol (2), and calcium content measurement by complexation of calcium with cresolphtalein complexone in an alkaline medium (3). Absorbance was examined spectrophotometrically at 580 nm.

### 2.8. X-ray Elemental Analysis of Calcium Ions in Apple Fruit at Tissue Level—Energy Dispersive X-ray Spectroscopy (EDS)-SEM

Cube-shaped fruit material was fixed in 2.5% glutaraldehyde and 2% paraformaldehyde in Phosphate Buffered Saline (PBS) and washed in MiliQ water. Samples were washed in saline solution and dehydrated in a series of alcohols (20%, 30%, 50%, 70%, 80%, 90%, 96%, and 99.8%). After dehydration drying in a critical point, the dryer system was carried out using liquid CO_2_ to withdraw water from the samples (CPD7501, Polaron Range, Germany). The selected spots of the fruit sample were analysed using energy dispersive X-ray spectroscopy (EDS) with a Bruker X-ray detector equipped with a scanning electron microscope with operating conditions of the electron microprobe of 20 kV (SEM, Zeiss Ultra Plus, Oberkochen, Germany). At least five replicates of X-ray line scans were obtained for each sample, and then data analyses were carried out according to Li [26].

### 2.9. Analysis of Results

The data were statistically analysed using OriginPro 8.5 software (Origin Lab v8.5 Pro, Northampton, MA, USA). For comparisons of the mean values, an analysis of variance (one-way ANOVA) followed by post hoc Tukey’s honestly significant difference test was used. For all analyses, the significance level was estimated at *p* < 0.05. A significant level of 1 indicates that the difference of the means is significant at the 0.05 level, and the means of the results are significantly different.

Representative image sets were selected for preparation of figures and edited using the CorelDrawX7 graphics program.

## 3. Results

### 3.1. Structure of AGPs

**Figure 1:**
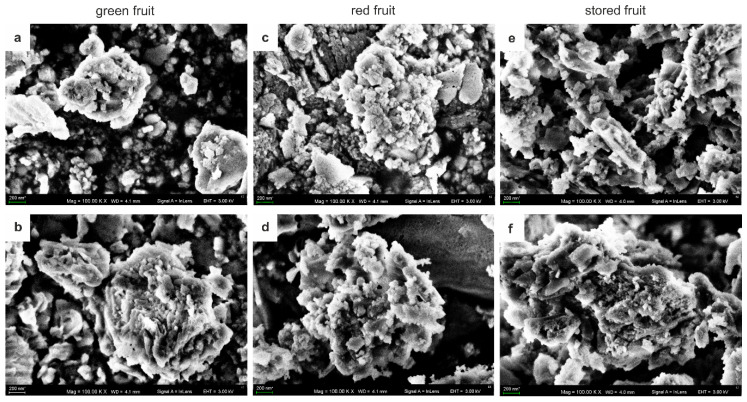
SEM microphotographs of AGPs extracted from fruit at different stages of ripening: green fruit (**a**,**b**), red fruit (**c**,**d**) and stored fruit (**e**,**f**). Bars: 200 nm.

Figure 1a–f shows SEM microphotographs of AGPs extracted from fruit at the different stages of ripening and freeze-dried. In each analysed sample, AGPs appeared as globular aggregated structures composed of a large number of variously shaped small-sized granules. The approximate diameter of the single granule was in the range of 100 to 200 nm, while their aggregates had a diameter of 2–3 µm in all examined samples regardless the ripening stage. One may conclude that aggregation of AGP after extraction does not depend on the developmental program.

Unlike SEM imaging, the HPLC analysis of AGP composition revealed clear differences along ripening time. A characteristic common feature of AGPs at each stage is the high proportion of carbohydrates. Table 1 shows the monosaccharide and uronic acids (UA) composition expressed in mol %. AGPs contained predominantly Ara, Gal, Glc, GalA, and Xyl. Other compounds, including Fuc, Rha, Man, and GlcA, were present in lower amounts. The distribution of monosaccharides and UA was heterogeneous at the ripening stages. AGPs isolated at early stage of development (green fruit) contained substantially higher amounts of Gal (30.5 mol %) than the other samples (15.2 mol % and 16.5 mol % for ‘red’ and ‘stored’ apples, respectively). In turn, the Ara content was distinctly lower in the AGPs in the green fruit (14.6 mol %) than at two other stages (>24.4 mol %). It is clear that the amount of Ara increased progressively along ripening reaching 34.9 mol % after 1 month of storage. The content of Glc was similar in the AGPs from the green (22.3 mol %) and red (21.6 mol %) fruit, and significantly lower in the stored fruit (6.3 mol %). The AGPs from the green fruit contained a visibly lower amount of GalA (7.3 mol %) than the AGPs from the red (19.4 mol %) and stored fruit (20.2 mol %).

**Figure 2:**
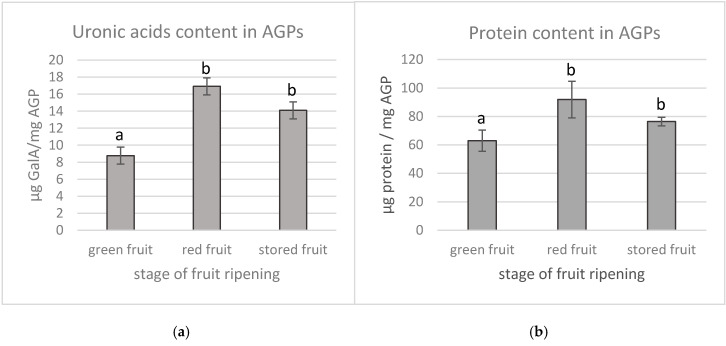
Qualitative analysis of uronic acids (**a**) and protein content (**b**) in AGPs extracted from green, red and stored apple fruit. Results are expressed in µg per mg of AGPs. Different letters indicate significant differences among the development stages (according to ANOVA with *p* < 0.05).

The uronic acids content was examined also with the continuous flow analysis (CFA) technique (Figure 2a). The colorimetric determination of UA in AGP samples from fruit at the different stages of ripening confirmed HPLC analyses. Despite using different analytical methods, the AGPs extracted from the green fruit contained a significantly a lower amount of UA (8.8 ± 1.5 µg/mg) than the AGPs extracted from the red (16.9 ± 1.6 µg/mg) and stored fruit (14.1 ± 1.4 µg/mg).

The protein content was measured using the Bradford method. Similarly, the AGPs extracted from the green fruit contained significantly lower proteins per mg of AGPs (62.96 ± 7.4 µg), representing 3.15% of the sample. In comparison, the protein moiety of the AGPs extracted from the red fruit represented 4.58% (91.9 ± 12.86 µg/mg) and 3.8% (76.39 ± 3.07 µg/mg) in the case of the stored fruit AGPs (Figure 2b).

### 3.2. Calcium Content in AGPs and Calcium Distribution in Fruit during the Ripening Process

**Figure 3:**
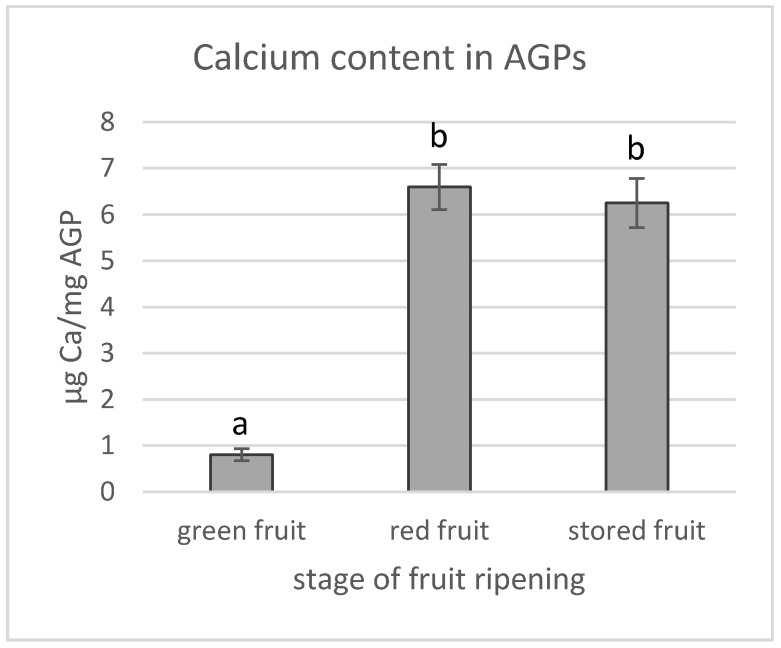
Qualitative analysis of calcium content in AGPs extracted from green, red and stored apple fruit (**a**). Results are expressed in µg of calcium per mg of AGPs. Different letters indicate significant differences among the development stages (according to ANOVA with *p* < 0.05).

The calcium content in all examined AGP samples was also determined using the colorimetric method with a continuous flow analyser (Figure 3). Similar to the previous measurements the lowest amount of calcium (0.8 ± 0.1 µg/mg) was found in the AGPs extracted from the green fruit. During ripening, the calcium amount in the AGPs increased by 8-fold just for the full red ripe stage, reaching a value of 6.6 ± 0.5 µg/mg that remained almost unchanged during storage.

**Figure 4:**
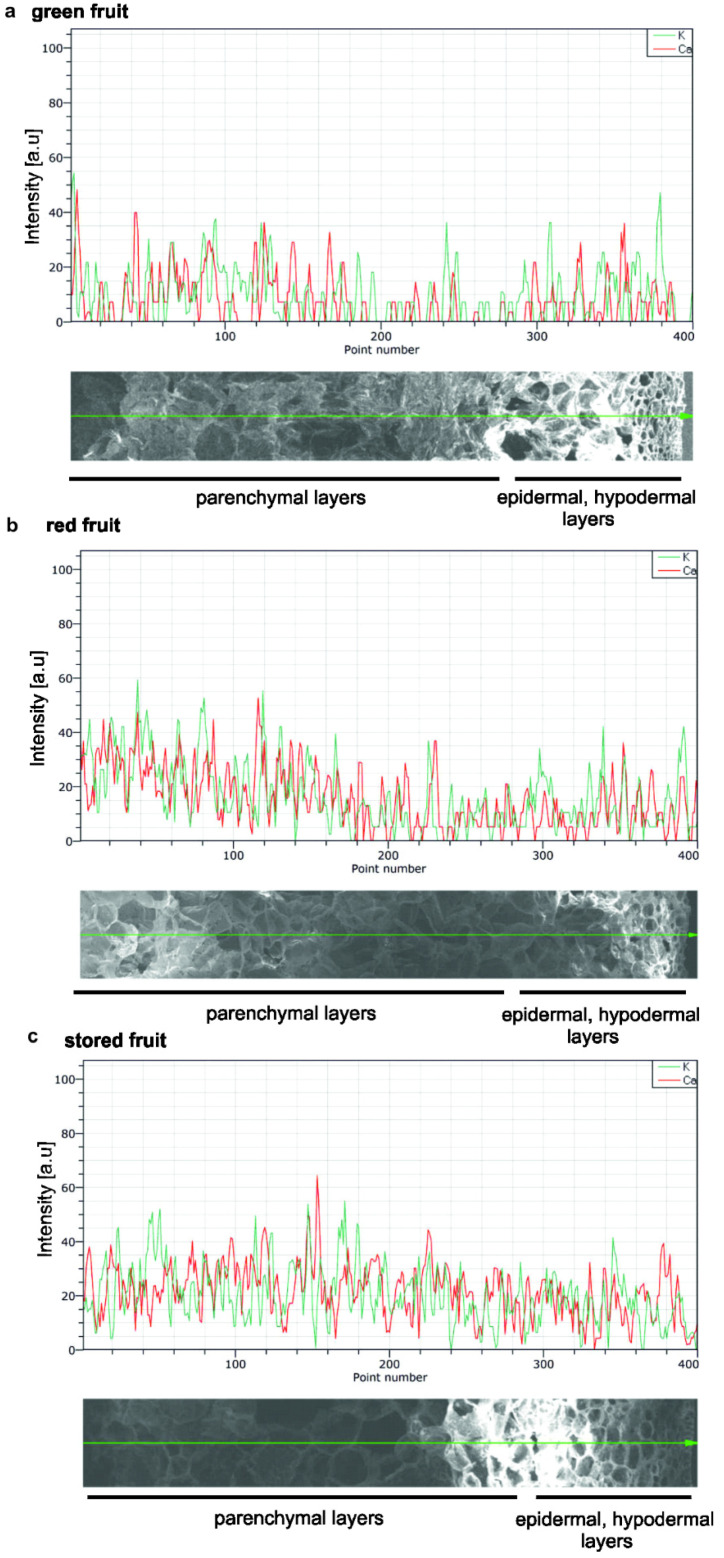
Mapping of calcium and potassium ion distribution in apple fruit at different stages of ripening: green fruit (**a**), red fruit (**b**) and stored fruit (**c**). Studies on Ca content was detected by SEM X-ray line profiles with magnification 100 ×. Red curve, Ca intensity counts. Green curve, K intensity counts. Green line on SEM images, the scanning line.

The occurrence of calcium ions in fruit in situ was analysed using elemental mapping by scanning electron microscopy–energy dispersive X-ray spectrometry (SEM-EDS) at the tissue level. The investigations of the spatial distribution of calcium in fruit focused on calcium mapping in epidermal, hypodermal, and parenchymal tissues. Figure 4a–c shows temporal distribution of calcium and potassium in the fruit tissue at the three stages of ripening. The X-ray analysis identified the presence of calcium through elemental mapping; however, the spectra showed that calcium on the surface of fruit was regularly arranged in all examined tissues, and the Ca signal was detected in all epidermal, hypodermal, and parenchymal tissues. Moreover, as shown by the SEM X-ray line profiles, there was no heterogeneous distribution of Ca in the fruit at the different stages of ripening (Figure 4a–c). Furthermore, the pattern of potassium distribution obtained demonstrated similar content of both macronutrients in the apple tissue.

## 4. Discussion

Research on extraction of plant components with functional and economic significance has been conducted for many years since fruit are a great source of polysaccharides, e.g., pectins with pro-health properties, industrial applications, and economic importance [27]. Arabinogalactan proteins are one of the commonly occurring proteoglycans in the cell walls. The first information about the possibility of extraction of AGPs from fruit and their characterisation was published 20 years ago [6]. The pioneering studies demonstrated that AGPs isolated from *Lycium chinense* fruit tissue were mainly composed of sugar chains with a strictly defined composition characterised by an overwhelming presence of Ara and Gal at a ratio of 3:1 with a trace amount of Fuc, Xyl, Man, Glc, and GalA [6]. The structural properties of AGPs in fruit were in agreement with earlier reports of AGPs extracted from different plant organs, in which the carbohydrate moiety constituted nearly 95% of the whole molecular mass of the AGP particle [1]. The composition of AGPs extracted from apple fruit revealed similar features to mentioned above, i.e., presence of Gal, Ara, GalA, and Glc that were the dominant sugars and smaller amounts of Rha, Man, and Fuc. Moreover, our study has demonstrated that the content of sugar components in the AGPs from the apple fruit considerably changes during the fruit ripening process, which may play a role in the function of the AGP complex for fruit tissue.

Overall, the structural properties, including the occurrence of carbohydrate units, exert an effect on other AGP features. Transmission electron microscopy imaging provided knowledge of the molecular shape of AGPs. One type of the shape models, i.e., the “wattle blossom”, shows that the polysaccharide chains are folded into globular units to generate a spheroidal shape. In turn, the “twisted hairy rope” model demonstrates that the polysaccharide chains wrap around the rod-like core protein [1]. Additionally, analyses of AGP monomers revealed a strong tendency to self-assemble into higher-order structures. The aggregates were composed of small, ellipsoidal building blocks [28]. The aggregates of AGPs were observed in *Nicotiana tabacum* [29] and in *Arabidopsis thaliana* [30]. The capacity of AGPs to agglomerate was connected with an adhesive ability to form proper cohesive strength in sticky plant exudates in *Hedera helix* [31], in gum Arabic from *Acacia senegal* [32], and in charophytes [33]. The present morphological analyses using SEM imaging showed that AGPs extracted from the fruit were globular particles with the ability to aggregate into larger structures. Moreover, it should be emphasised that SEM provides only qualitative estimation of the surface structure. We may only assume that the shape and aggregation are also correlated with the carbohydrate composition of AGPs. A high number of monosaccharides forming the carbohydrate chains in the AGP glycan moiety facilitates the formation of new side branches and additional intermolecular interactions.

The functional importance of the extensive glycosylation of AGPs is emphasised in studies on mutants with modified expression of prolyl 4 hydroxylases (P4Hs), which catalyse proline hydroxylation during the AGP glycan synthesis process [34]. Suppression of expression of tomato P4Hs leads to structural alterations of the AGPs’ glycan moiety, causes a delay in over-ripe fruit abscission [34], and changes in cell expansion by the distribution of other cell wall components [35]. Furthermore, AGPs act as signalling molecules possessing a pH-dependent Ca^2+^-binding glycomotif. The abundance of Ca^2+^-binding subunits allows them to play role as a periplasmic Ca^2+^ capacitor. Given this property, AGPs are involved in most aspects of plant development, i.e., auxin and extension growth, tropisms and mechanotransduction, intracellular dynamics, wound response, flowering, fertilisation, and early embryogenesis [15,16]. In the case of fruit, the concentration of calcium ions is related to tissue firmness, and changes in the Ca^2+^ level correspond with intercellular debonding to cell wall rupturing, an increase in intercellular spaces, and changes in the fruit tissue structure due to pectin–calcium cross-links [36]. Metabolic changes in Ca^2+^ in the cell wall and in the apoplast are related to cell wall and plasma membrane stability and integrity. Alterations in the Ca^2+^ content in these compartments may be related to Ca^2+^ depletion and may weaken plasma membrane structures, leading to cell death and thus Ca^2+^ deficiency symptoms in the whole fruit tissue [17]. The total Ca content in the fruit at harvest is usually sufficient for good quality, and cellular Ca partitioning and distribution within fruit tissues often lead to localised deficiencies in the fruit [37,38]. In our study, the monosaccharide content of AGPs extracted from the fruit varied depending on the stages of ripening. We detected significant changes in the content of uronic acids, which was confirmed by the colorimetric and HPLC analyses. During the ripening process, the level of galacturonic acid was three times higher in the AGPs in the red fruit in comparison to the AGPs in the green fruit. Moreover, galacturonic acid units, i.e., the main components of pectic polysaccharides, have the ability to interact with divalent calcium cations [39]. In the present study, the quantitative analyses of the Ca content in AGPs showed that the Ca amount also increased with the progress of the ripening process. This should be underlined, as the increase was maximally eight-fold higher in the AGPs extracted from the red fruit than in the AGPs extracted from the green fruit. The ability of calcium accumulation by AGP molecules can be connected with the increasing content of uronic acids, mainly GalA, during the ripening process. Galacturonic acid participates in the formation of complexes with Ca^2+^ and other divalent ions through its carboxyl and hydroxyl groups according to the egg-box model [40]. Such cross-linking of Ca^2+^ significantly influences viscosity of GalA solutions and may affect the structural properties of plant cell wall constituents [41]. In line with the existing knowledge, our data confirmed the typical carbohydrate composition of AGPs and may be the basis for studies regarding their presumed properties of binding calcium ions. In the present study, X-ray analysis, which is used for identification of elemental composition, showed that all tissues forming the fruit structure have similar concentrations of calcium during various phases of ripening. The obtained results underline that calcium localisation is related with the binding by cell wall components and, thus, mentioned changes may be observed only at the subcellular level.

As described in our previous papers, the distribution of AGPs is closely correlated with spatial modifications of the cell wall–plasma membrane continuum during ripening [42]. The immunocytochemical approach used to examine changes in the fruit cell wall at the cellular and subcellular levels showed that the examined proteoglycans occurred in different zones, depending on alterations in the cell wall–plasma membrane. Furthermore, the localisation of AGPs was found to depend on the condition of the cell wall–plasma membrane changing during the postharvest storage as a result of senescence process. Various investigations primarily show changes in the aforementioned pattern as a presumed mechanism of plant adaptation in response to environmental stress factors, such as fungal infection [43,44,45]. Moreover, super-resolution imaging with Stimulated Emission Depletion Microscopy (STED), combined with immunocytochemical analyses used to visualise the dynamic behaviour of AGPs, has also shown that AGPs are extracellular cargo receptors and migrate across the plasma membrane to initiate endocytosis [46]. Unfortunately, the mechanism of the changes in the spatio-temporal distribution of AGPs is yet unknown. Taken together, in the present study we proved that the structure of AGPs is closely related with the fruit ripening process. The structural changes of AGPs are similar to those observed in our previous studies on AGP glycan epitopes and confirm the ongoing metabolism of their carbohydrate moiety during the progress of the fruit maturation and ripening process. We may postulate that changes in AGP glycosylation during ripening process have an impact on the fruit quality by exerting an effect on ion binding, on the establishment of cell wall–plasma membrane integrity, and on cross-linking with other cell wall constituents.

## 5. Conclusions

The application of biochemical methods for structural investigations throughout the fruit ripening process is an indispensable part of AGP studies. These preliminary studies on the extraction and structural properties of AGPs allow an assumption that AGPs might play a role in determining the fruit cell wall structure in apples and perhaps in other fruits. Work is now in progress to determine whether there are changes in the cell wall features in fruit with and without normal function of AGPs, which is investigated using related mutants. Moreover, the present results open up new possibilities for the analysis of AGPs as proteoglycans with the ability affect calcium in fruit. These results can be the basis for further research. Therefore, any approach to modifying the cell wall calcium-binding capacity should be monitored closely.

## Figures and Tables

**Table 1:** Monosaccharide composition of AGPs (mol %) extracted from fruit at different stages of ripening.

	Monosaccharide Composition (mol%)								
	Man	Rha	GlcA	GalA	Glc	Gal	Xyl	Ara	Fuc
AGPs from green fruit	2.9	1.8	3.9	7.3	22.3	30.5	12.8	14.6	3.8
AGPs from red fruit	2.7	3.8	1.7	19.4	21.6	15.2	9.8	24.4	1.5
AGPs from stored fruit	3.7	5.6	2.0	20.2	6.3	16.5	8.6	34.9	2.1
